# Food resource competition between African wild dogs and larger carnivores in an ecosystem with artificial water provision

**DOI:** 10.1002/ece3.11141

**Published:** 2024-03-17

**Authors:** Elisa Sandoval‐Serés, Moreangels Mbizah, Shepherd Phiri, Simbarashe Pride Chatikobo, Marion Valeix, Esther van der Meer, Egil Dröge, Daphine Madhlamoto, Hillary Madzikanda, Peter Blinston, Andrew J. Loveridge

**Affiliations:** ^1^ Wildlife Conservation Research Unit (WildCRU), Department of Biology, Recanati‐Kaplan Centre University of Oxford Tubney UK; ^2^ Painted Dog Conservation (PDC) Dete Zimbabwe; ^3^ Wildlife Conservation Action Belgravia, Harare Zimbabwe; ^4^ CNRS, Université de Lyon, Université de Lyon 1, Laboratoire de Biométrie et Biologie Evolutive, CNRS UMR Villeurbanne France; ^5^ CEFE, Univ Montpellier, CNRS, EPHE, IRD, Univ Paul Valéry Montpellier 3 Montpellier France; ^6^ Long‐Term Socio‐Ecological Research Site (LTSER) France, Zone Atelier ‘Hwange’ Hwange National Park Zimbabwe; ^7^ Zambian Carnivore Program Mfuwe Zambia; ^8^ Scientific Services Main Camp, Hwange National Park Zimbabwe Parks and Wildlife Management Authority (ZPWMA) Dete Zimbabwe

**Keywords:** carnivores, diet, interspecific competition, resource partitioning, waterholes

## Abstract

Predators of similar size often compete over prey. In semi‐arid ecosystems where water is a limiting resource, prey availability can be affected by water distribution, which further increases resource competition and exacerbate conflict among predators. This can have implications for carnivore dietary competition. Hence, we evaluated the dynamics of food resource competition between African wild dogs and four competing predators (cheetahs, leopards, lions and spotted hyaenas) in different seasons and across areas with different waterhole densities in Hwange National Park, Zimbabwe. We used the frequency of occurrence of prey items found in predators' scats to analyse diet composition, overlap and prey preference. For most predators, kudu was most frequently consumed and preferred. Low and medium water‐dependent prey (medium and small‐sized) were mostly consumed by wild dogs, leopards and cheetahs. Wild dog diet overlap was high with all predators, particularly with hyaenas and lions. There were no seasonal differences in the predators diet. The diet overlap of wild dogs with lions was highest in the low waterhole density area, and wild dog diet composition did not differ significantly from the diet of lions and hyaenas. In the low waterhole density area, wild dogs and hyaenas broadened their niche breadth, and predators diet had a higher proportion of low water‐dependent prey. A low density of waterholes increased food resource competition. However, high density of waterholes, where there is more prey availability, can increase the aggregation and density of predators, and hence, increase the risks involved in interspecific competition on wild dogs. To reduce food resource competition on wild dogs, we propose to conserve larger‐bodied prey that are less dependent on water (e.g. kudu, reedbuck, eland and gemsbok). As the use of water pumping is common practice, we propose maintaining water management heterogeneity where prey which is less dependent on water can also thrive.

## Graphical abstract

We evaluated the dynamics of food resource competition between African wild dogs and four competing predators (cheetahs, leopards, lions and spotted hyaenas) across areas with different waterhole densities (maximum, high and low) in Hwange National Park, Zimbabwe. We propose to conserve larger‐bodied prey not much dependent on water (e.g. kudu) by keeping their populations stable. We emphasise maintaining water management heterogeneity.

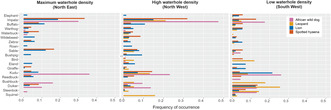

## 1 INTRODUCTION

Large predators help maintain stable ecological processes as they exert top‐down effects (Dalerum et al., 2008). Thus, their conservation is crucial to maintain healthy ecosystem functioning (Martinez‐del‐Rio et al., 2001). Among mammals, large predators (>21 kg) are a distinct functional group on top of the food chain that can feed on a wide range of prey sizes (Radloff & Du Toit, 2004). As large predators belong to the same carnivorous guild, their ecological niches overlap, which results in competition (Amarasekare, 2003; Radloff & Du Toit, 2004).

To promote coexistence among competing species, a form of niche separation is necessary; this can be temporal, spatial or through diet partitioning (Amarasekare, 2003). Diet separation can reduce exploitative competition, which is when species indirectly compete for common resources through depletion of these resources (Ghoddousi et al., 2017; Tilman, 1982). Thus, species need to adapt their foraging strategies to maximise their fitness (Schoener, 1971). As predators of similar size compete over prey (Cupples et al., 2011; Harihar et al., 2011), a subordinate predator (smaller in size) may change its diet due to the presence of a dominant predator (larger in size), especially when food is scarce (Hayward & Kerley, 2008; Mbizah et al., 2012). In such a situation, prey selection could depend more heavily on competition among predators than on predator–prey characteristics (Jones & Barmuta, 1998; Radloff & Du Toit, 2004).

Diet overlap serves as an indication of resource competition (Du Preez et al., 2017). A high degree of diet overlap, which can indicate the potential for a high level of resource competition, can exacerbate conflict among predators (Donadio & Buskirk, 2006; Du Preez et al., 2017; Fedriani et al., 2000). One way to reduce interspecific competition is through diet segregation, particularly when competing species overlap spatio‐temporally (Balme et al., 2017; De Almeida Jácomo et al., 2004; Gerber et al., 2012). Subordinate predators can reduce interspecific competition by feeding on different prey groups (e.g. prey water dependency or prey size) (Davis et al., 2018; Hayward & Kerley, 2005); as well as, through segregating their diet seasonally (Azevedo et al., 2006; Carvalho & Gomes, 2004; Jones & Barmuta, 1998) and spatially (different habitats and areas) (Jones & Barmuta, 2000; Tsunoda et al., 2019). However, it is possible that the options for diet segregation are reduced when prey abundance decreases, in which case subordinate predators will be affected more heavily than dominant ones (Creel et al., 2018; Ferretti et al., 2020; Schoener, 1971; Steinmetz et al., 2020).

The African wild dog (*Lycaon pictus*) (~22 kg) (referred to as wild dog throughout the manuscript) is an endangered, social and subordinate carnivore within Africa's large carnivore guild (IUCN, 2023). It suffers from interspecific competition with lions (*Panthera leo*) (150–250 kg) and spotted hyaenas (referred to as hyaenas throughout the manuscript) (*Crocuta crocuta*) (~70 kg). These two dominant predators affect wild dogs through direct killing, exploitative competition, exclusion from prey rich areas and kleptoparasitism (Creel, 2001; Van der Meer et al., 2011; Van der Meer, Rasmussen, & Muvengwi, 2013b; Vanak et al., 2013). Wild dog diet overlaps not only with the diet of lions and hyaenas, but also with the diet of leopards (*Panthera pardus*) (23–31 kg) and cheetahs (*Acinonyx jubatus*) (34–64 kg) (Hayward & Kerley, 2008; Mbizah et al., 2012).

In arid and semi‐arid ecosystems, water becomes a limiting resource in the dry season and is, therefore, in some areas, actively pumped to provide water to animals (Owen‐Smith, 1996). Variation in water availability affects the abundance and distribution of herbivores (Redfern et al., 2003; Valeix, 2011), which in turn affects the abundance and distribution of predators (Valeix et al., 2010, 2012), and ultimately, the level of intraguild competition between predators (Périquet et al., 2021). The widespread use of artificially supplied water in African savannahs (Edwards et al., 2015; Owen‐Smith, 1996; Sutherland et al., 2018) can impact carnivore interactions and potentially affect the fate of endangered species such as wild dogs. Although food competition between large African predators has been widely studied (Creel et al., 2018; Hayward & Kerley, 2008; Mbizah et al., 2012), the role of water on the dynamics of this competition has never been assessed.

Here, we aim to identify the level of food competition (diet composition, diet overlap and prey preference) between wild dogs and the four competitive predators (cheetahs, leopards, lions and spotted hyaenas) in different seasons (weather seasons: wet‐early dry [November–June]/late dry [July–October]; wild dogs' behavioural seasons: nomadic (non‐breeding) [September–April]/breeding (restricted in movement due to denning) [May–August]) across areas characterised by contrasting water availability to assess the role of water availability, and hence provisioning, on the potential exploitative competition between wild dogs and other large carnivores. As water can have an impact on prey species distribution and abundance, our main hypothesis is that food resource competition between wild dogs with larger predators would differ between seasons and between areas with different waterhole densities due to differences in prey distribution and availability. We predict that the potential for resource competition, that is diet overlap, is higher between wild dogs and the other predators during the dry and breeding season and in areas with a lower density of waterholes, as prey would likely be less available there.

## 2 METHODS

### 2.1 Study site

**FIGURE 1:**
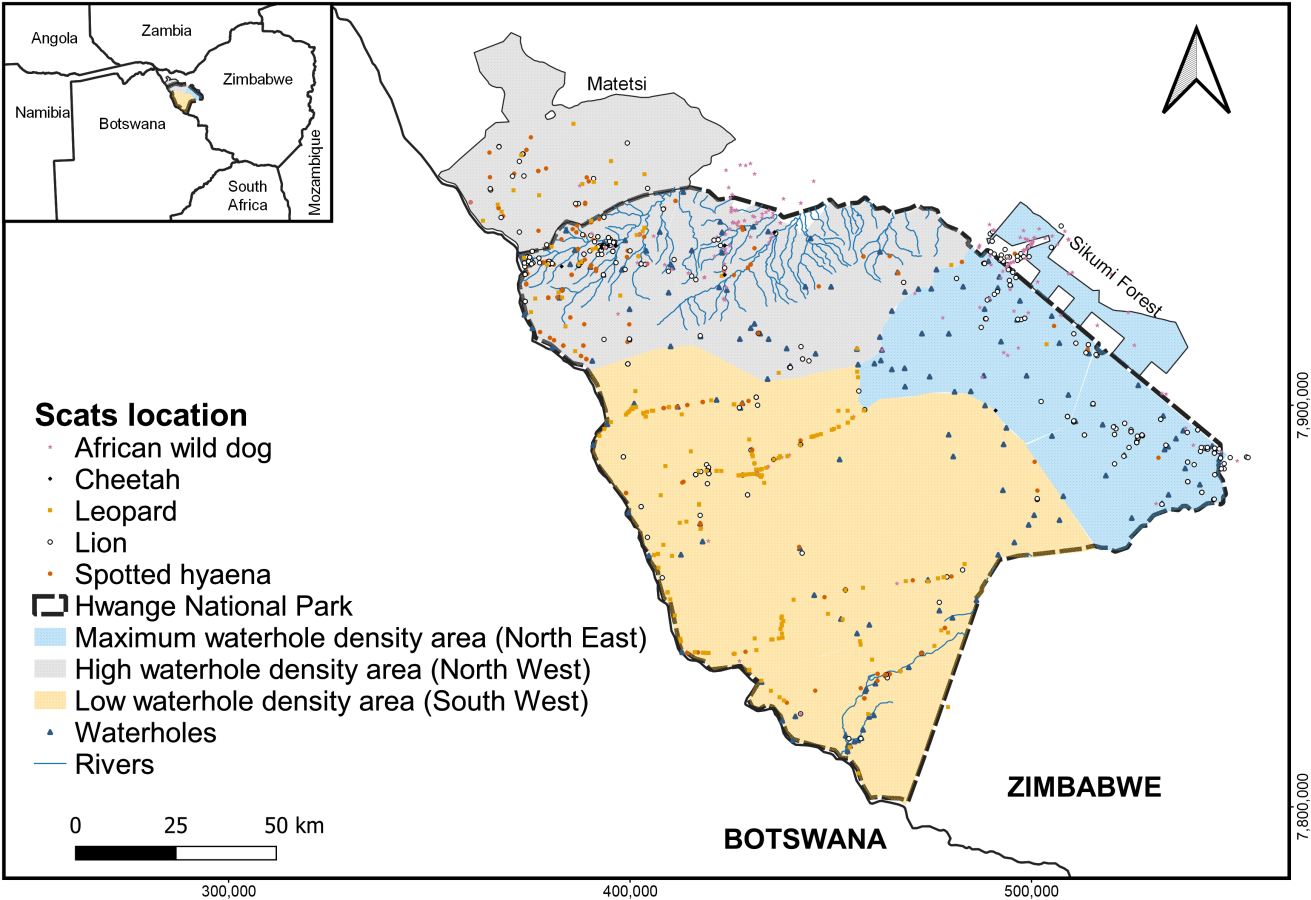
Map of the study area, Hwange National Park and adjacent areas, Zimbabwe, with locations of scat samples per predator within the three areas of the park with contrasting waterhole densities.

The study site is situated in Hwange National Park (HNP), an unfenced protected area without human settlements or paved roads and used for photographic tourism. The park covers ca. 15,000 km^2^ in western Zimbabwe (19:00′ S, 26:30′ E) (Figure 1), with altitudes between 800 and 1100 m. The habitat comprises of woodland, bushland and open areas of grassland mainly associated with waterholes (Arraut et al., 2018). HNP does not have natural perennial water sources; thus, in the dry season, animals depend on artificially provisioned waterholes. The wet‐early dry season (November–June) has a mean rainfall of ~540 mm, and the late dry season (July–October) has a mean rainfall of ~12 mm (Wilderness Safaris Zimbabwe, unpublished data for 2010–2017). During the late dry season, deciduous trees lose their foliage, and pasture is of the lowest quality. Waterholes are mainly found in the northern area of HNP (both in the North West (NW) and North East (NE) areas—Figure 1—where waterhole density is 2.0 and 4.6 per 100 km^2^, respectively—Table 1). The NW area has the most fertile soil (basalt soil) and is characterised by woodland and bushland; while woodland and open grassland in Kalahari sands characterise the NE area. Moreover, in the NW area there are rivers that carry water during the wet season (Chamaillé‐Jammes et al., 2007). The South West (SW) area is the driest part of the park (waterhole density is 0.5 per 100 km^2^; Table 1) and characterised by bushy grassland on Kalahari sand soil with almost no water provisioning during the dry season (Arraut et al., 2018; Rogers, 1993). Areas adjacent to HNP include human settlements, trophy hunting areas (e.g. Matetsi) and photographic safari areas (Loveridge et al., 2017). During the study period in HNP (2013–2019), the density of dominant predators was estimated to be: ~2.9 (±2.2 sd) lions/100 km^2^ and ~10.7 (±6.1 sd) hyaenas/100 km^2^ (Table 1; Loveridge et al., 2022).

**TABLE 1:** Characteristics of the study areas in Hwange National Park and surroundings, Zimbabwe.

Areas		North east	North west	South west
Waterhole density during the dry season per 100 km^2^		3.25	~1.4 + seasonal rivers	~0.2
Vegetation types and soil		Woodland, open grassland, bushed grassland on Kalahari sand soil.	Woodland, grassland and bushland on basalt soil (most fertile).	Bushed grassland on Kalahari sand soil.
Average abundance of African wild dogs (2014–2020) (±SE standard error)		Pack size = ~7.5 (±0.6) Pack number = ~10.0 (±1.3)	Pack size = ~10.9 (±1.0) Pack number = ~7.9 (±1.3)	Pack size = ~8.3 (±2.3) Pack number = ~2.2 (±0.7)
Average densities (2013–2019) per 100 km^2^ (±SD standard deviation)	Leopard	~2.3 (±1.0)	~3.0 (±1.5)	~2.0 (±0.1)
	Lion	~2.3 (±1.3)	~6.5 (±0.3)	~1.8 (±1.1)
	Spotted hyaena	~8.4 (±2.1)	~19.1 (±5.4)	~6.1 (±1.1)
Summary of prey species abundances^a^		Highest for: duiker, steenbok, wildebeest, sable, kudu, elephant and zebra.	Highest for: impala, buffalo, bushbuck, kudu, warthog.	Low, except for gemsbok, bushpig and reedbuck.

*Note*: Vegetation and soil taken from Arraut et al. (2018). African wild dogs' abundance including pups less than 1‐year‐old (PDC Annual Reports). Predator densities taken from Loveridge et al. (2022).

^a^ Prey density and relative abundance index taken from Tables S3 and S4.

### 2.2 Data collection

Faecal samples of cheetahs, leopards, lions and hyaenas were collected opportunistically along roads, trails, kills and latrines from 2012 until 2015. Faecal samples for wild dogs were collected while following packs from 2012 to 2020. The identification of the faeces of predators was based on morphology, colour, odour and associated tracks (Mbizah et al., 2012) or by directly observing defecation. Following Mbizah et al. (2012), samples were photographed, washed in acetone, dehydrated in 100% ethanol and dried on filter paper. For prey species identification, 6–8 hairs from the washed hair sample were selected from different parts of the prey species' pelage. Hair cross‐sections and scale pattern imprints made on wood glue were photographed through a microscope and each hair was identified to species level using photographic reference guides (Buys & Keogh, 1984; Kent, 2004; Seiler, 2010; Taru & Backwell, 2014). To limit the probability of pseudo‐replication, for lion and hyaena scats, we considered only one sample collected per 24 h per location (location was considered the same if ≤1 km apart). For wild dog's scats, we considered only one sample collected per 24 h from the same pack, regardless of location. We acknowledge that there could still be some pseudo‐replication by only considering 24 h, as complete digestion can take longer, however, in order to keep a good sample size we used 24 h based on Mbizah et al. (2012).

To calculate prey abundance, we used data from line‐transect surveys carried out in the north of HNP in September/October (late dry season) and in May/June (early dry season) each year from 2012 until 2019. Further details of line‐transect survey methodology in HNP can be found in Chamaillé‐Jammes et al. (2009). A total of 492 camera trap stations (Cuddeback models 1125, 1149 and C1, Non‐ Typical, WI, USA; Panthera V4, Panthera, NY, USA; Stealthcam G42NG, Grand Praire, TX, USA) were deployed between 2013 and 2020 across nine surveyed sectors (three surveyed sectors per area) (total effort: 23,319 trap days). Camera trap stations were placed along trails or roads and spaced in a grid of 5 km apart (Figure 1) (Loveridge et al., 2022).

### 2.3 Analyses

#### 2.3.1 Diet composition and comparisons

To determine the diet of each predator, we analysed the faecal data as a whole (including all samples: ‘All’ category), as well as, per area (NW, NE and SW areas) and per season over all years. One limitation of dividing the park into three areas was that we were not able to control for instances where consumption and defecation took place in different areas. However, scats collection at the margin between these areas was minimal (Figure 1). For the seasonal analyses, we took the following categories into account: ‘wet‐early dry’ season (November–June) ‘late dry’ season (July–October); and ‘nomadic’ season (September–April) when wild dogs are nomadic versus ‘breeding’ season (May–August) when wild dogs are denning and, therefore, restricted in their movement (packs that were not denning during the breeding season were excluded from this analysis: 12 scats). We categorised prey species by level of water dependency (high, medium, low) and diet (mixed feeder [browser and grazer], browser, grassland grazer, woodland grazer, omnivorous, carnivore, other) (based on Redfern et al., 2003; De Boer et al., 2010; Hayward & Hayward, 2012; Table S1). In cases where we found carnivore species in the diet, they were included in the analyses because even though they can be killed as part of interspecific competition, they are also sometimes preyed upon by predators (Breuer & Breuer, 2005; Du Preez et al., 2017; Rasmussen, 1996). We used prey size based on mean female weight as described by Cumming and Cumming (2003) and Kingdon (2004) (XS extra‐small <5 kg, S small 6–24 kg, M medium 25–99 kg, L large 100–349 kg, XL extra‐large >350 kg) (Balme et al., 2017; Mbizah et al., 2012) (Table S1). Wild dogs hunt together as a pack which allows them to increase hunting efficiency and to hunt for larger prey (Creel & Creel, 1998). To determine if there was any correlation of wild dog pack size with prey size, we performed a Cumulative Link Mixed Model—CLMM (*ordinal* package; Christensen, 2022), using prey size as the dependent variable, pack name as a random factor and pack hunting size (excluding pups) as a fixed factor. As the distribution of prey weights was clumped, we used prey weight class, a categorical variable and hence used ordinal regression: CLMM.

To determine if we collected the minimum number of scats needed to adequately describe the diet of predators, we calculated prey species accumulation curves using the function *specaccum* in the *vegan* package (Oksanen et al., 2018) (Figure S1). Because of a small sample size (<21) neither the cheetah diet (information only available for the NW area) nor leopard diet in NE and seasonally were included in statistical comparisons, as we did not perform any analysis when there were less than 21 scats within a category (Table S2a).

For each area and season, we calculated the frequency of occurrence (Klare et al., 2011). For the ‘All’ category, we also estimated the relative biomass intake. Following Woodroffe et al. (2007), we used the Weaver's equation derived from the grey wolf (*Canis lupus*): prey mass per scat (kg) = 0.439 + (0:008 * prey species' mean female weight) to estimate the biomass intake for wild dogs (Weaver, 1993). Following Briers‐Louw (2017) and Du Preez et al. (2017), we used the Ackerman's equation derived from pumas (*Puma concolor*) for the other predators: prey mass per scat (kg) = 1.980 + (0.035* prey species' mean female weight) (Ackerman et al., 1984). As these formulas are not specific for the species of our study, the biomass results are only indicative representations of the proportions of biomass consumed and not necessarily the accurate biomass value. When calculating biomass, we corrected for the maximum stomach capacity of each predator, which were corrected at a maximum of 10 kg for leopards, 24 kg for hyaenas and 50 kg for lions (Bertram, 1975; Kruuk, 1972). As the highest biomass consumed per scat for wild dogs and cheetahs (4 kg) did not exceed maximum stomach capacity (~9 kg) (Creel & Creel, 1995), there was no need to correct their biomass calculations. For seasonal and spatial comparisons on predators diet composition, we performed a permutational analysis of variance (PERMANOVA) of prey species found in the predators' scats with 1000 permutations and controlling for ‘year’ using the *adonis2* function of the *vegan* package (Oksanen et al., 2018). We used PERMANOVA analysis because it is a non‐parametric test that compares groups' differences where it is possible to stratify the permutations performed (Oksanen et al., 2018). This meant that we were able to control for ‘year’ in our comparisons. When comparing prey species composition, we used the Jaccard index (presence/absence data: prey species in each scat): $J=\frac{A+B-2J}{\left(A+B-J\right)}$; where *A* and *B* are the numbers of species in compared predator scats, and *J* is the number of species shared in predator scats (Jaccard, 1908). When comparing prey categories (prey water dependency, prey diet and prey size), we used the Bray–Curtis dissimilarity index (using abundance data, as more than one prey species could be found in each category): $=\frac{\sum_{i=1}^{n}\;\left|X_{\mathrm{ij}}-X_{\mathrm{ik}}\right|}{\sum_{i=1}^{n}\left(X_{\mathrm{ij}}+X_{\mathrm{ik}}\right)}$; where $X_{\mathrm{ij}}$ and $X_{\mathrm{ik}}$ are the numbers of prey species per category *i* found in predator scats *j* and *k; n* is the number of categories (Bray & Curtis, 1957).

As this is a study using scats and not direct observations, we were not able to determine the proportion of prey consumed through scavenging or hunting; however, it is mainly lions and hyaenas that scavenge or kleptoparasitize if the opportunity appears, wild dogs very rarely scavenge (Creel & Creel, 2002; Périquet et al., 2015).

#### 2.3.2 Diet overlap and niche breadth

To determine the diet overlap of wild dogs with the other predators, we used Pianka's index (Pianka, 1973): $O_{\mathrm{jk}}=\frac{\sum_{i}^{n}p_{\mathrm{ij}}\;p_{\mathrm{ik}}}{\sqrt{\sum_{i}^{n}p_{\mathrm{ij}}^{2}\sum_{i}^{n}p_{\mathrm{ik}}^{2}}}$; where $O_{\mathrm{jk}}$ is the diet overlap between predators *j* and *k*; $p_{\mathrm{ij}}$ is the prey proportion *i* of the total prey used by predator *j*; $p_{\mathrm{ik}}$ is the prey proportion *i* of the total prey used by predator *k*; and *n* is the total number of prey items. This index ranges between 0 (no overlap) and 1 (complete overlap). For seasonal diet overlap, we only included the samples collected in the northern part of HNP, as in the SW no wild dog samples were collected in the wet‐early dry seasons and there was no breeding information on wild dogs available. We evaluated statistical significance of Pianka's index with a null model in which diet items are reshuffled randomly and independently (with 10,000 iterations) while maintaining the observed prey species richness. For this, we used the *EcoSimR* package (Gotelli et al., 2015). To determine the diet niche breadth of each of the three predators, we used the standardised Levin's index (Krebs, 1999; Levins, 1968): $B=\frac{1}{\sum p^{2}}$ and $\mathrm{Bs}=\frac{B-1}{n-1}$, where B is niche breadth; p is the proportion of prey items; Bs is standardised niche breadth; and *n* is the total number of prey. Both diet overlap and niche breath indexes were calculated taking into account all consumed prey by all predators.

#### 2.3.3 Prey preference

To determine whether prey consumption was based on prey availability or prey preference, we used Jacobs' index: $D=\left(r-p\right)/\left(r+p-2\mathrm{rp}\right)$, where r is the proportion of prey in the diet and p is the proportion of prey available. A Jacobs' index value of −1 indicates maximum avoidance and a value of +1 maximum preference (Jacobs, 1974). We calculated Jacobs' index using two different measurements for prey availability (that we calculated): (1) prey density and (2) prey relative abundance index (RAI). Prey density is a more accurate measure for prey abundance; however, RAI was also used, as prey density was not available in the SW of HNP. To coincide with the sampling period of scat collection of predators, we calculated prey abundance data from all years (2012–2019) to calculate prey preference of wild dogs, and prey abundance data of 2012–2015 to calculate prey preference of the other predators. To calculate prey density, we used distance sampling methods (Buckland et al., 2001) using the *Distance* package (Miller, 2020). We used 5% truncation, and ran models using half‐normal, uniform and hazard‐rate key‐functions with cosine/polynomial series expansion, both including and excluding vegetation type as a covariate for detection function. We selected the model with the smallest Akaike Information Criterion (Burnham & Anderson, 2002) and checked the goodness of fit with a chi‐square test (results of *p*‐value were above 0.20). To calculate prey RAI, we used camera trap data and calculated RAI as follows: independent records/trap days. We used as independent records, consecutive photographs of different individuals (appearing on the same picture together) of the same species taken more than 30 min apart (O'Brien et al., 2003). We calculated RAI indexes per survey sector and then averaged the indexes per area. Prey densities can be found in Table S3; and prey RAI can be found in Table S4. We considered that there was statistical evidence of a difference when a *p*‐value was over 0.05; and we performed all our analyses using R 4.1.2 (R Core Team, 2022).

## 3 RESULTS

### 3.1 Diet composition and comparisons

In total, for wild dogs, there were 225 food items sampled in 209 scats with 20 prey species identified; for cheetahs, 27 items in 26 scats and 7 species; for leopards 246 items in 204 scats and 25 species; for lions there were 351 items in 342 scats and 33 species; and for hyaenas there were 337 items in 317 scats and 33 species (Table S2a). Main results are summarised in Table 2.

**TABLE 2:** Predators diet results summary.

	Maximum waterhole density area	High waterhole density area	Low waterhole density area
African wild dog main prey	Kudu, bushbuck and waterbuck.	**Impala** and bushpig.	**Duiker** and **steenbok.**
Cheetah main prey	No data.	Bushbuck, duiker and scrub hare.	No data.
Leopard main prey	No data.	**Impala,** kudu and squirrel.	**Duiker,** bushbuck, **steenbok** and birds.
Lion main prey	Impala, buffalo and sable.	Buffalo and bushpig.	Kudu and **duiker**.
Spotted hyaena main prey	Impala and sable.	Kudu, **impala** and wildebeest.	**Duiker** and **steenbok.**
Diet overlap of African wild dogs with lions	0.59	0.63	**0.77**
Diet overlap of African wild dogs with spotted hyaenas	0.60	**0.88**	0.68

*Note*: Main prey refers to most common and preferred. In bold = shared prey species between African wild dogs and other predators per area and the highest diet overlap.

There was no evidence of seasonal differences (either in wet‐early dry vs. late dry [pseudo‐*F*_2,839_ = 1.14, *p* = .59], or in nomadic vs. breeding; [pseudo‐*F*_1_,_815_ = 0.64, *p* = .16]) in the diet of wild dogs, lions and hyaenas (Table S5a). However, there was evidence that predators diet differed in different areas of the park (Table 3; Table S5b).

**TABLE 3:** Differences on prey water dependency in the diet of predators in areas with contrasting waterhole densities in Hwange National Park, Zimbabwe.

	Maximum versus high waterhole density area	High versus low waterhole density area	Maximum versus low waterhole density area
African wild dog	pseudo‐*F*_1_,_167_ = 4.65 ***p* = .0012** ^**a**^ *r* ^2^ = .027	pseudo‐*F*_1_,_99_ = 4.35 *p* = .51 *r* ^2^ = .042	pseudo‐*F*_1_,_108_ = 0.92 *p* = .25 *r* ^2^ = .008
Leopard	NA	pseudo‐*F*_1_,_199_ = 14.56 *p* = .26 *r* ^2^ = .068	NA
Lion	pseudo‐*F*_1_,_285_ = 0.41 *p* = .62 *r* ^2^ = .0014	pseudo‐*F*_1_,_191_ = 19.74 ***p* = .012** ^a^ *r* ^2^ = .094	pseudo‐*F*_1_,_202_ = 23.23 ***p* = .048** ^a^ *r* ^2^ = .103
Spotted hyaena	pseudo‐*F*_1_,_205_ = 1.32 *p* = .34 *r* ^2^ = .0064	pseudo‐*F*_1_,_131_ = 19.74 *p* = .081 *r* ^2^ = .094	pseudo‐*F*_1_,_292_ = 17.73 ***p* = .002** ^a^ *r* ^2^ = .057

Abbreviation: NA, not applicable due to lack of data.

^a^ In bold = statistical evidence for significant results (*p* < .05).

**FIGURE 2:**
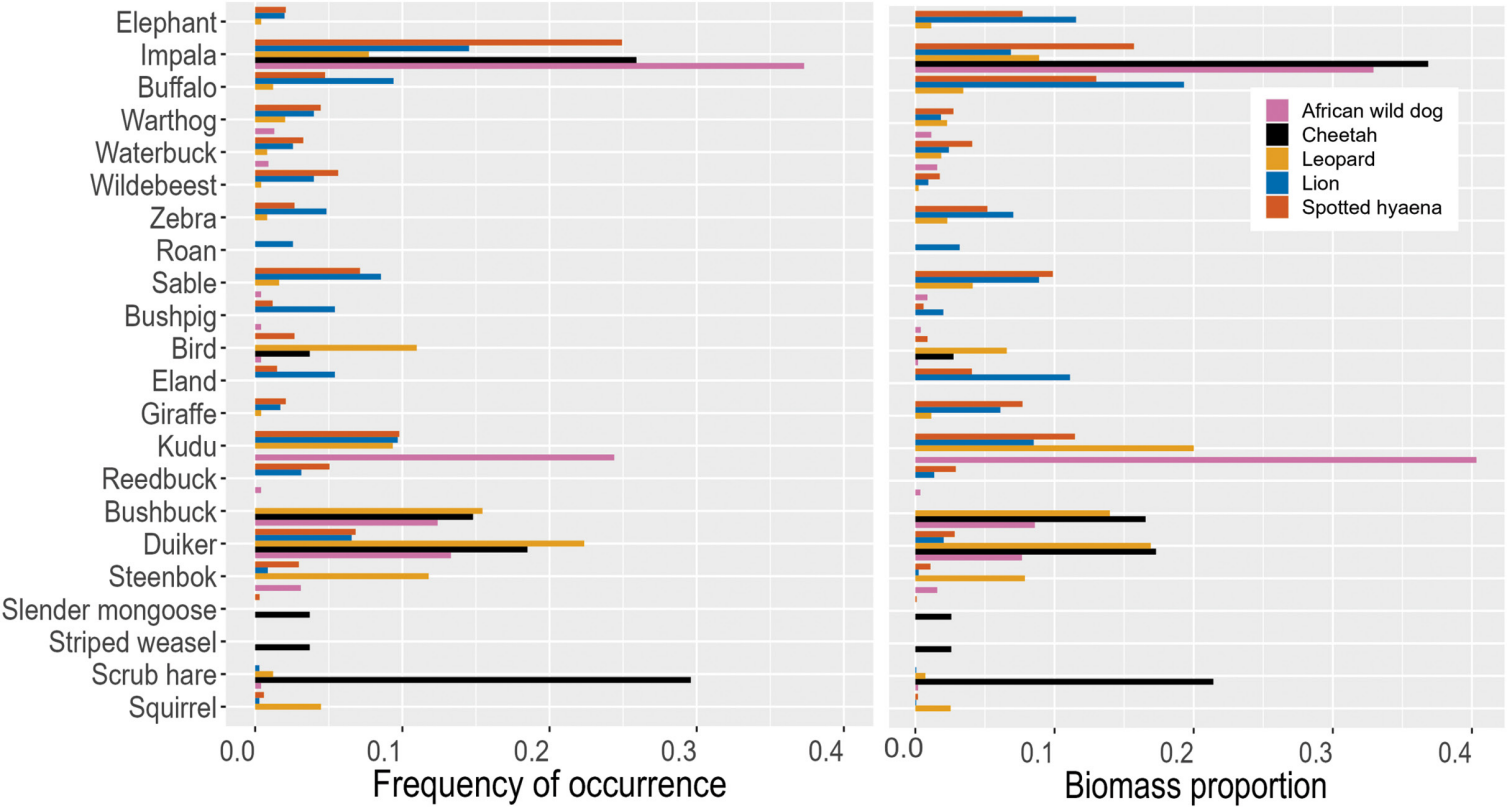
Frequency of occurrence and biomass proportion of prey species for the five large carnivores in Hwange National Park, Zimbabwe. Only including species with either frequency of occurrence or biomass larger than 0.03. Figure S2 includes all species. Order from high water‐dependent prey to low water‐dependent prey.

Overall, the most frequent prey species (by occurrence) for wild dogs were impala (*Aepyceros melampus*), kudu (*Tragelaphus strepsiceros*), duiker (*Sylvicapra grimmia*) and bushbuck (*Tragelaphus scriptus*) (~94% of total diet; where kudu and impala encompassed ~66% of the diet). In terms of biomass, kudu was the prey species with the highest contribution to the wild dog diet (~40%) followed by impala (~33%). For cheetahs, the most common prey were scrub hare (*Lepus saxatilis*), impala, duiker (*Sylvicapra grimmia*) and bushbuck (~80%), and impala the most important in terms of biomass (~37%). For leopards, the most common prey were duiker, bushbuck and steenbok (*Raphicerus campestris*) (~51%) and kudu the most important in terms of biomass (~20%). For lions, impala, kudu, buffalo (*Syncerus caffer*) and sable (*Hippotragus niger*) were the most common prey species (~42%), while the prey with the highest biomass contributions were buffalo, eland (*Taurotragus oryx*) and elephant (*Loxodonta africana*) (~42%). For hyaenas, the most important prey species both in terms of frequency and in terms of biomass were impala, kudu and sable (~42%); and in addition to these species, buffalo was important in terms of biomass (~16%) (Figure 2; Figure S2).

Only in the low waterhole density area there was no statistical evidence that wild dog diet differed from the diet of both lions and hyaenas, this was consistent in terms of diet composition (lion: pseudo‐*F*_1,74_ = 1.079, *p* = .33; hyaena: pseudo‐*F*_1,129_ = 1.56, *p* = .068), in terms of prey water dependency (lion: pseudo‐*F*_1,483_ = 6.91, *p* = .70; hyaena: pseudo‐*F*_1,461_ = 7.91, *p* = .25) and prey size (lion: pseudo‐*F*_1,74_ = 1.54, *p* = .21; hyaena: pseudo‐*F*_1,129_ = 1.62, *p* = .20). Contrarily, in the high waterhole density area, wild dog diet differed significantly from the diet of the other three predators in terms of diet composition, prey diet and prey size, but not in prey water dependency. Leopard diet composition was different from the diet of wild dogs in the areas tested (NW and SW), except in terms of prey diet and prey size in the low waterhole density area (Table 4, Tables S5b and S6).

**TABLE 4:** Differences between the diet of African wild dogs with the diet of other predators in Hwange National Park, Zimbabwe.

	African wild dog versus		
	Maximum waterhole density area	High waterhole density area	Low waterhole density area
Leopard	NA	pseudo‐*F*_1_,_113_ = 5.66 ***p* = .0019** ^a^ *r* ^2^ = .048	pseudo‐*F*_1_,_185_ = 3.15 ***p* = .0059** ^a^ *r* ^2^ = .017
Lion	pseudo‐*F*_1_,_236_ = 12.77 ***p* < .001** ^a^ *r* ^2^ = .051	pseudo‐*F*_1_,_216_ = 13.89 ***p* < .001** ^a^ *r* ^2^ = .06	pseudo‐*F*_1_,_74_ = 1.079 *p* = .33 *r* ^2^ = .014
Spotted hyaena	pseudo‐*F*_1_,_110_ = 4.96 *p* = .24 *r* ^2^ = .043	pseudo‐*F*_1_,_262_ = 6.99 ***p* < .001** ^a^ *r* ^2^ = .026	pseudo‐*F*_1_,_129_ = 1.56 *p* = .072 *r* ^2^ = .012

Abbreviation: NA, not applicable due to lack of data.

^a^ In bold = statistical evidence for significant results (*p* < .05).

**FIGURE 3:**
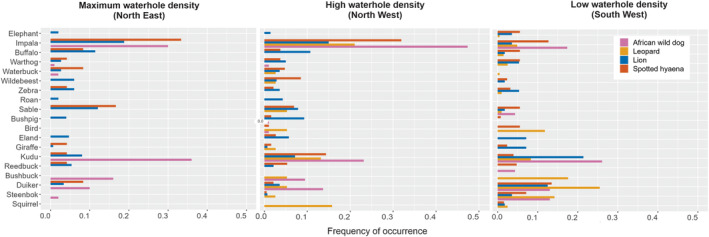
Frequency of occurrence per area of the proportions of the main prey in the diet of four predators in three different areas of Hwange National Park, Zimbabwe. Prey species are ordered by size. Only including species with the frequency of occurrence larger than 0.03. Figure S3 includes all species. Order from high water‐dependent prey to low water‐dependent prey.

Wild dog diet differed significantly between the high waterhole density area (NW) and the maximum waterhole density area (NE) in composition (pseudo‐*F*_1,167_ = 4.14, *p* = .004), prey water dependency (pseudo‐*F*_1,167_ = 4.65, *p* = .0012) and prey size (pseudo‐*F*_1,167_ = 4.61, *p* = .015) (Tables S5b and S6). Wild dogs had a higher proportion of impala (medium mixed feeder) in their diet in the maximum waterhole density area (NE), a higher proportion of kudu (large browser) in the high waterhole density area (NW), and a more diverse diet in the low waterhole density area (SW), including duiker and steenbok (Figure 3). For lions there was some evidence that they had differences in their diet between the different areas (*p* = .057), for the other predators there was no evidence for differences in their diet in the different areas (Table S5b). However, when comparing prey water dependency in their diet, lions and hyaenas had a higher proportion of less water‐dependent prey in the low waterhole density area (lion: pseudo‐*F*_1,191_ = 19.74, *p* = .012; hyaena: pseudo‐*F*_1,292_ = 17.73, *p* = .002), such as kudu and duiker for lion diet and duiker for hyaena diet (Table 3, Figure 3; Figure S3, Tables S7 and S8).

Overall, wild dogs had a higher frequency of high and low water‐dependent species (which also are medium mixed and browsing feeders) in their diet. We did not find any correlation of wild dog pack size with prey size (estimate = −0.02 (SE = 0.027); *p* = .45). Cheetahs and leopards consumed the highest proportion of low water‐dependent prey species (which also are small and medium mixed and browser species); whereas lions and hyaenas consumed a high frequency of water‐dependent species (which also are large and medium grassland grazers and mixed feeders) in their diet (Figure S4).

### 3.2 Diet overlap and niche breadth

**FIGURE 4:**
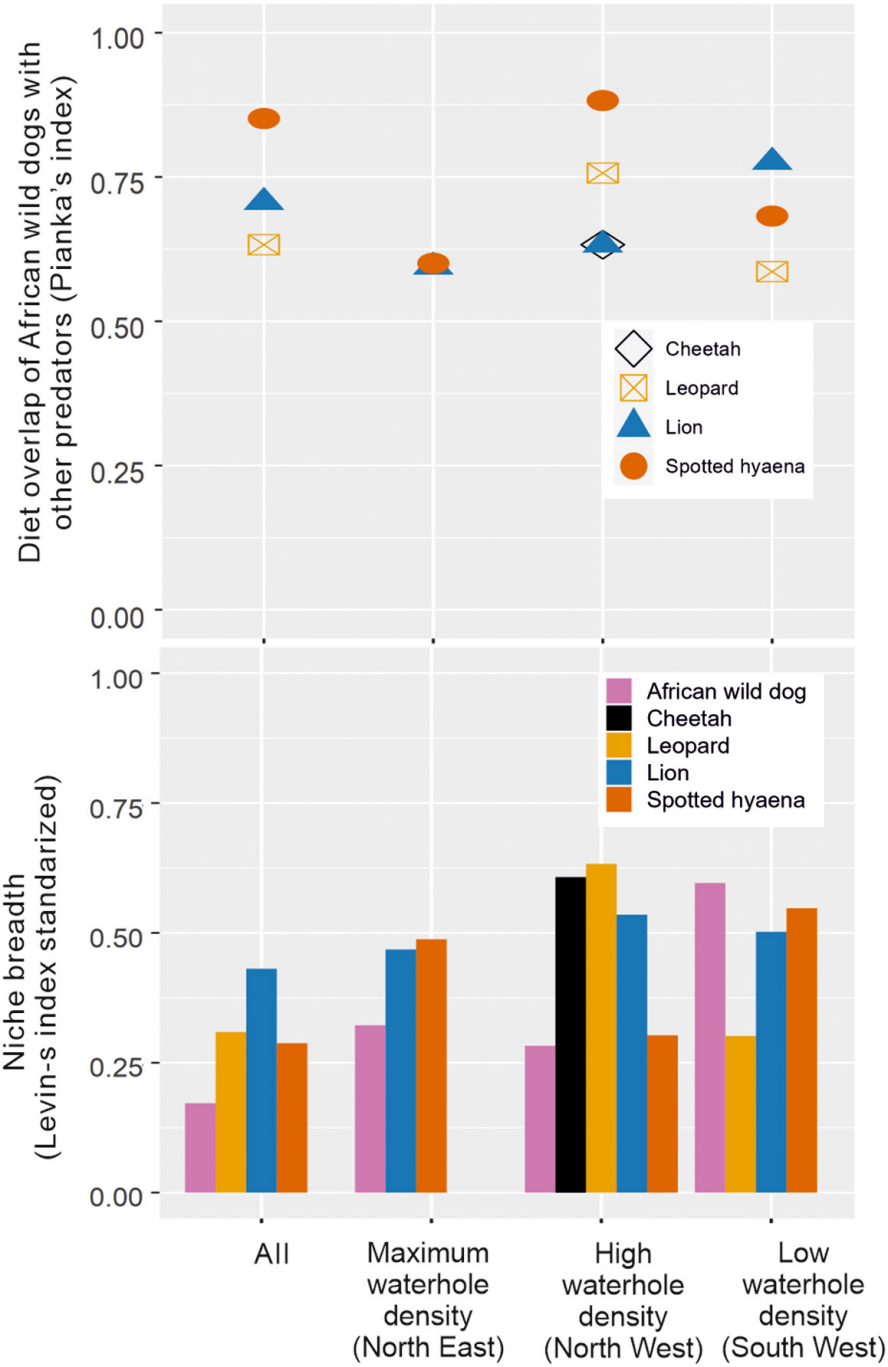
Diet overlap and dietary niche breadth of five predators in three different areas of Hwange National Park, Zimbabwe. Indexes are calculated including carnivores in the predators diet. Data on cheetahs only in North West, no data of leopards in North East.

In total, wild dog diet overlap was high with all predators (>0.55), but higher with hyaenas (0.85) and lions (0.71) (Table S2b; Figure 4). There was more diet overlap between wild dogs and predators in the high and low waterhole density areas than in the maximum waterhole density area. In the low waterhole density area, wild dog and lion diet overlapped the most, while in the high waterhole density area wild dog and hyaena diet overlapped the most (Figure 4). All Pianka's indices were significantly different from null models (*p* < .05). Overall, wild dogs had the narrowest niche breadth of the five predators. However, in the low waterhole density area, wild dogs had the broadest diet niche (Figure 4).

### 3.3 Prey preference

**FIGURE 5:**
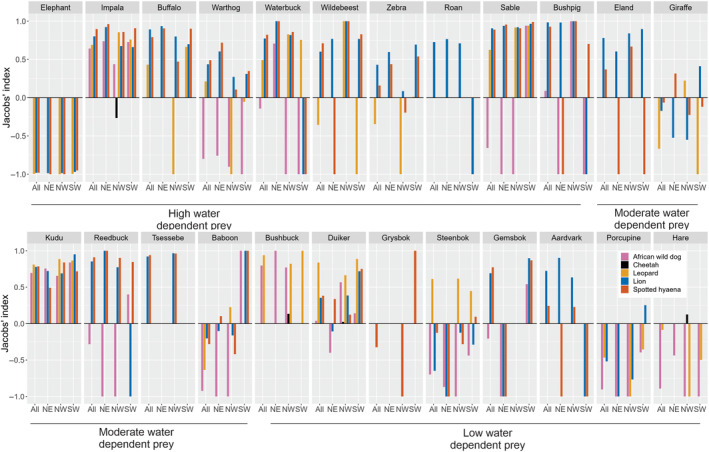
Diet preference (Jacobs' Index calculated with RAI: relative abundance index) of five predators in three different areas of Hwange National Park, Zimbabwe. NE = North East (Maximum waterhole density area); NW = North West (High waterhole density area); SW = South West (Low waterhole density area). Figure S5 has the diet preference with Jacobs' Index calculated with prey density.

Kudu (medium water‐dependent prey), duiker and bushbuck (low water‐dependent prey) were preferred by wild dogs, cheetahs and leopards in all areas. In addition, leopards preferred impala, steenbok, sable, waterbuck (*Kobus ellipsiprymnus*), wildebeest (*Connochaetes taurinus*) and giraffe (*Giraffa camelopardalis*). Lions and hyaenas preferred duiker, wildebeest, waterbuck, sable, eland, reedbuck (*Redunca arundinum*) and warthog (*Phacochoerus africanus*); and hyaenas also preferred kudu and giraffe. Only in the maximum waterhole density area wild dogs preferred waterbuck. In general, prey density and prey RAI gave similar results. Prey RAI results showed that most prey were less abundant in the low waterhole density area (Table 1, Table S4a). However, when we calculated Jacobs' index using prey density, buffalo was not preferred, and impala was only preferred in the maximum waterhole density area; but when using prey RAI both prey species were preferred (density values were higher than RAI values for both species) (Figure 5; Figure S5).

## 4 DISCUSSION

Artificial water provisioning in arid and semi‐arid ecosystems is a common practice throughout Africa (Edwards et al., 2015; Owen‐Smith, 1996; Sutherland et al., 2018). As water distribution can affect herbivores availability and abundance (Redfern et al., 2003; Valeix, 2011), the competition level of predators over prey can also be affected. In HNP, competition over prey was high between wild dogs and larger predators (cheetahs, leopards, lions, hyaenas), and the levels of food resource competition between wild dogs with dominant predators (lions and hyaenas) differed between areas with different waterhole densities associated to different levels of resource availability.

### 4.1 Diet composition

Wild dog diet overlap was high with all predators. Overall, it was highest with hyaenas, similar to findings from Breuer and Breuer (2005) and Mbizah et al. (2012), and lowest with leopard and cheetah diet. This contrasts with other studies where wild dog diet overlapped more with cheetah and leopard diet than with lion and hyaena diet (Hayward & Kerley, 2008; Vogel et al., 2019). This might indicate that wild dogs are subjected to higher levels of dietary competition with the two most dominant predators (lions and hyaenas) in HNP than in other parts of Africa. This could add competition pressure of dominant predators to wild dogs in HNP.

Wild dogs, cheetahs and leopards preyed more upon less water‐dependent species (which are mixed, browser, medium and smaller species). While, lions and hyaenas preyed more frequently upon high water‐dependent species (which are grazers and larger sized species). These differences in prey categories could facilitate coexistence.

Contrary to our predictions that food resource competition was going to be different between seasons, season did not influence diet composition of predators nor the level of resource competition between wild dogs with lions and hyaenas. In HNP, Davidson et al. (2013), also found that overall there were no seasonal differences in lion diet. In other carnivores and ecosystems, there were seasonal foraging differences that depended on seasonal food availability (Lanszki et al., 2020; Padial et al., 2002; Vissia et al., 2022). Our results demonstrate that the level of resource competition did not depend on prey fluctuation due to seasonality, as waterholes are also pumped in the dry season. Perhaps, instead of seeking different prey in different seasons, wild dogs focused on different age and sex classes of the same species (Pole, 2000), depending on the breeding season of their main prey.

The main prey of wild dogs were impala, kudu, duiker and bushbuck. These results are consistent with other studies in HNP (Childes, 1988; Van der Meer, Rasmussen, & Muvengwi, 2013b) and other areas (Hayward et al., 2006; Mbizah et al., 2012; Pole et al., 2004). However, impala was not always preferred by wild dogs, but consumed according to availability. Prey size tends to increase with wild dog pack size (Creel & Creel, 1995), but we did not find a correlation with pack size and prey size. This result could be because we did not consider prey age classes, or because even only one individual wild dog can kill large prey species such as a kudu (PDC unpublished data). In densely vegetated areas, wild dogs hunting success is higher (Creel & Creel, 2002), and the risk of kleptoparasitism is lower (Creel & Creel, 1996, 1998). Therefore, wild dogs may prefer to make their kills in dense vegetation where kudus, bushbucks and duikers (as strict browsers) are commonly found (Valeix et al., 2009).

### 4.2 Resource competition in areas with different waterhole densities

In the low waterhole density area, the level of resource competition of wild dogs with lions and hyaenas was higher. This was mainly due to both dominant predators shifting their diet (lion and hyaena diet had a higher proportion of wild dogs preferred prey species in this area). Only in this area, wild dogs diet did not differ from lion and hyaena diet, nor did it differ in terms of prey diet and size with leopard diet (browsers were consumed more by the four predators). Browsers are less dependent on water than grazers (Redfern et al., 2003; Valeix, 2011), which could also explain why browsers were consumed more in the low waterhole density area. In this area, wild dogs and leopards had a higher proportion of duiker and steenbok in their diet; hyaenas consumed proportionally more duikers compared to other areas; and lions consumed a significantly higher proportion of low water‐dependent species (kudu, duiker, steenbok) than in other areas of HNP. For both dominant predators, in the driest area, there is less abundance of preferred prey, which are mainly large sized, grazers and high water‐dependent prey; this can explain why lions and hyaenas consume more prey preferred by wild dogs (prey smaller in size and less water‐dependent) in that area. All this potentially indicates a high level of resource competition between wild dogs with both dominant predators (especially with lions) in the area with the lowest waterhole density.

Wild dogs had the largest niche breadth in the low waterhole density area. In this area, wild dogs preferred prey included a wider range of prey species (i.e. sable, reedbuck, gemsbok [*Oryx gazelle*] and baboon [*Papio ursinus*]), which were avoided in the high and maximum‐density areas, possibly because these species are more dangerous to hunt (Van der Meer et al., 2019). Wild dogs would tend to avoid hunting these species to avoid any potential fitness costs imposed by hunting dangerous prey. Carnivores need to become more generalist when there is lower prey abundance (Lanszki et al., 2019; Macarthur & Levins, 1967), especially subordinate carnivores competing with dominant ones for food (Dröge et al., 2017; Petroelje et al., 2021). Consequently, wild dogs might have had to broaden their niche breadth in this area to compensate for a high diet overlap with the dominant predators, as well as, due to a lower relative prey abundance.

### 4.3 Conservation implications

It is important to conserve complete predator guilds to preserve ecological processes (Dalerum et al., 2008). Although wild dogs are adapted to coexist with other predators as they have evolved with them for millennia (Turner, 1990), water provisioning could potentially aggravate the interspecific competition of wild dogs by reducing areas to escape competition inside protected areas with high dominant predator densities and outside protected areas with high anthropogenic threats (Van der Meer, Fritz, et al., 2013a).

Wild dogs might not necessarily need an exclusive prey species to survive, as kudu, impala and duiker are also important prey for the four other predators, and bushbuck is a species also preferred by leopards and cheetahs. However, a reduction of prey abundance can increase food resource competition (Creel et al., 2018; Karanth & Sunquist, 1995; Sévêque et al., 2020), which is what seems to be happening in the low waterhole density area in HNP.

Although wild dogs do not seem to be limited by prey availability (Creel & Creel, 1998; Woodroffe et al., 2007), reducing prey availability can affect wild dogs by increasing intraguild competition (Creel et al., 2018). Moreover, low prey abundance could affect wild dogs' reproduction (Marneweck, Druce, & Somers, 2019b), potentially increase intraspecific competition between African wild dog packs (Marneweck, Marneweck, et al., 2019a) and increase the probability of packs consuming livestock herewith provoking conflict with humans (Woodroffe et al., 2005).

It is crucial to conserve both density and diversity of prey, especially prey preferred by threatened predators (Davidson et al., 2019; Hayward & Kerley, 2008). Kudu is an important species because it was preferred and had frequency and high biomass contribution in the diet of most predators (especially in wild dog diet). In the low waterhole density area with high resource competition, and where lions and hyaenas were consuming a higher proportion of smaller prey less dependent on water (possibly because there was less abundance of large bodied prey), conserving large sized prey preferred, like eland and gemsbok, by lions and hyaenas, would most likely decrease the food competition on wild dogs. Hence, we emphasise not only to prioritise the conservation of kudu, but also the conservation of other large prey species moderately dependent on water, such as reedbuck, eland and gemsbok, mainly in areas with low waterhole density. This is consistent with Creel et al., 2018 who found that a lack of large bodied prey leads to more dietary competition. Hence, we propose to conserve these prey species by keeping their populations stable but not necessarily increasing their abundance. To prioritise the conservation of these prey species we recommend to avoid the culling of them, as well as to have enough spaces without too many waterholes: either by closing waterholes in the maximum waterhole density area, or by not creating more waterholes in areas with high waterhole densities.

In high waterhole density areas, there is a higher density of dominant predators (Loveridge et al., 2022), which means that there is a higher number of competitors for food in those areas. Moreover, if prey abundance is high (such as in high waterhole density areas), dominant carnivores' density can increase (Carbone & Gittleman, 2002; Hayward et al., 2007) and have negative effects on wild dogs (Creel & Creel, 1996, 1998), such as excluding them from prey rich areas (Creel, 2001), or even through direct mortality (Prugh & Sivy, 2020). When food resource competition is high, diet partitioning might not play a major role in predators' niche segregation for coexistence. Instead, in those cases, spatiotemporal dimensions might be the main mechanisms allowing coexistence, such as wild dogs hunting in crepuscular times and dominant predators hunting at night, or by wild dogs avoiding areas highly used by lions (Bruno et al., 2003; Dröge et al., 2017; Tsunoda et al., 2019; Vissia et al., 2022).

Low waterhole density in the ecosystem increases food resource competition (especially with lions); but high waterhole density in the ecosystem (where there is more prey availability), can increase the density of predators (Macdonald, 2016), and hence, increase the risks involved in interspecific competition on wild dogs (Creel, 2001). Thus, we emphasise the need to maintain heterogeneity in water management actions.

## 5 CONCLUSION

Resource competition between wild dogs with larger predators, driven by fluctuations of prey availability and abundance, differed between areas with different waterhole densities, but not between seasons. Dietary competition of wild dogs with dominant predators (especially with lions) was highest in the low waterhole density area. To reduce food resource competition (exploitative competition) on wild dogs, we propose to conserve larger‐bodied prey that are less dependent on water. As food resource competition was high between wild dogs with the four larger predators, spatiotemporal partitioning might be playing a major role to allow coexistence.

## AUTHOR CONTRIBUTIONS

**Elisa Sandoval‐Serés:** Conceptualization (lead); data curation (lead); formal analysis (lead); funding acquisition (equal); methodology (equal); writing – original draft (lead); writing – review and editing (lead). **Moreangels Mbizah:** Data curation (equal); funding acquisition (equal); methodology (equal); resources (equal); writing – review and editing (supporting). **Shepherd Phiri:** Data curation (equal); writing – review and editing (supporting). **Simbarashe Pride Chatikobo:** Data curation (equal); writing – review and editing (supporting). **Marion Valeix:** Conceptualization (equal); methodology (equal); supervision (equal); validation (equal); writing – review and editing (equal). **Esther van der Meer:** Conceptualization (equal); methodology (equal); supervision (equal); validation (equal); writing – review and editing (equal). **Egil Dröge:** Conceptualization (equal); methodology (equal); supervision (equal); validation (equal); writing – review and editing (equal). **Daphine Madhlamoto:** Validation (equal); writing – review and editing (supporting). **Hillary Madzikanda:** Methodology (equal); project administration (equal); validation (equal); writing – review and editing (supporting). **Peter Blinston:** Funding acquisition (lead); project administration (lead); resources (lead); validation (equal); writing – review and editing (supporting). **Andrew J. Loveridge:** Conceptualization (equal); resources (equal); supervision (lead); validation (equal); writing – review and editing (equal).

## FUNDING INFORMATION

PDC, CONACYT and Universidad de Guadalajara (Mexico), Rufford Foundation, WildCRU funded this study.

## CONFLICT OF INTEREST STATEMENT

None declared.

## Supporting information


Data S1.

## Data Availability

The data is available in: https://figshare.com/articles/dataset/Sandoval_Seres_et_al_2024_Food_resource_competition_between_African_wild_dogs_and_larger_carnivores_in_an_ecosystem_with_artificial_water_provision_csv/25112936. doi: 10.6084/m9.figshare.25112936.
