# Supplementary material for: Food resource competition between African wild dogs and larger carnivores in an ecosystem with artificial water provision
Source: Ecol Evol. 2024 Mar 17;14(3):e11141. doi: 10.1002/ece3.11141 (PMC10944706; doi:10.1002/ece3.11141)
Supplement: Supplementary file 1 — Data S1. [file ECE3-14-e11141-s001.zip › Sandoval_Seres_Metadata.docx]

**Metadata**

Frequency of occurrence by item found in scats of predators.

First row: the species of predator where the scat belong.

First column: Prey found in scats of predators.

Other columns named by (season or area of the park):

Nomadic season: when African wild dogs were not breeding (September-April). Breeding season: when African wild dogs have their breeding season (May-August).

Late dry season (July-October).

Wet-early dry season (November-June).

Area of the park: NE: North East. NW: North West. SW: South West.
