# Supplementary material for: Food resource competition between African wild dogs and larger carnivores in an ecosystem with artificial water provision
Source: Ecol Evol. 2024 Mar 17;14(3):e11141. doi: 10.1002/ece3.11141 (PMC10944706; doi:10.1002/ece3.11141)
Supplement: Supplementary file 1 — Data S1. [file ECE3-14-e11141-s001.zip › Supporting information_clean.docx]

**Supporting information**

| **Table A1. Prey categories in the diet of African wild dogs, lions and spotted hyaenas in Hwange National Park, Zimbabwe.** | | | | | | |
| --- | --- | --- | --- | --- | --- | --- |
| **Prey (Common name)** | **Prey (Scientific name)** | **Prey water dependency** | **Prey diet** | **Adult female weight (kg)** | **Prey size** | **In predators diet**  AWD = African wild dog.  CH = Cheetah.  LE = Leopard.  LI = Lion.  SH = Spotted hyaena. |
| Aardvark | *Orycteropus afer* | Low | Other | 50 | M | LI SH |
| African wild dog | *Lycaon pictus* | Low | Carnivore | 23 | S | LI SH |
| Baboon | *Papio ursinus* | Moderate | Omnivorous | 21 | S | AWD LI SH |
| Banded mongoose | *Mungos mungo* | Low | Carnivore | 1.8 | XS | LI SH |
| Bat-eared fox | *Otocyon megalotis* | Low | Carnivore | 4 | XS | AWD LE |
| Bird |  | High | Other | 1 | XS | AWD CH LE SH |
| Buffalo | *Syncerus caffer* | High | Grassland grazers | 450 | XL | LE LI SH |
| Bushbuck | *Tragelaphus scriptus* | Low | Mixed | 35 | M | AWD CH |
| Bushpig | *Potamochoerus larvatus* | High | Omnivorous | 59 | M | AWD LE LI SH |
| Caracal | *Caracal caracal* | Low | Carnivore | 10.5 | S | LE LI |
| Cheetah | *Acinonyx jubatus* | Low | Carnivore | 50 | M | LE LI SH |
| Common genet | *Genetta genetta* | Low | Carnivore | 1.5 | XS | LE |
| Domestic cow | *Bos taurus* | High | Grassland grazers | 306 | L | AWD SH |
| Domestic sheep | *Ovis aries* | High | Grassland grazers | 42 | M | LE LI |
| Duiker | *Sylvicapra grimmia* | Low | Browser | 20 | S | AWD CH LI SH |
| **Prey (Common name)** | **Prey (Scientific name)** | **Prey water dependency** | **Prey group/diet** | **Adult Female weight (kg)** | **Prey size** | **In predators diet** |
| Eland | *Taurotragus oryx* | Moderate | Mixed | 450 | XL | LE LI SH |
| Elephant | *Loxodonta africana* | High | Mixed | 2275 | XL | LI SH |
| Fox |  | Low | Carnivore | 4 | XS | AWD |
| Gemsbok | *Oryx gazella* | Low | Woodland grazers | 225 | L | AWD LE LI SH |
| Giraffe | *Giraffa camelopardalis* | Moderate | Browser | 825 | XL | LE LI SH |
| Honey badger | *Mellivora capensis* | Low | Carnivore | 11.5 | S | LE SH |
| Impala | *Aepyceros melampus* | High | Mixed | 60 | M | AWD CH LI SH |
| Jackal |  | Low | Carnivore | 9 | S | SH |
| Klipspringer | *Oreotragus oreotragus* | Low | Mixed | 13.2 | S | AWD LI |
| Kudu | *Tragelaphus strepsiceros* | Moderate | Browser | 160 | L | AWD LE LI SH |
| Marsh mongoose | *Atilax paludinosus* | Low | Carnivore | 3.7 | XS | LE LI SH |
| Mice |  | Unknown | Other | 0.5 | XS | AWD LE SH |
| Pangolin | *Smutsia temminckii* | Low | Other | 9 | S | LE |
| Porcupine | *Hystrix africaeaustralis* | Low | Other | 17 | S | AWD LI |
| Reedbuck | *Redunca arundinum* | Moderate | Grassland grazers | 50 | M | AWD LI SH |
| Roan | *Hippotragus equinus* | High | Woodland grazers | 250 | L | LI |
| Sable | *Hippotragus niger* | High | Woodland grazers | 200 | L | AWD LE LI SH |
| **Prey (Common name)** | **Prey (Scientific name)** | **Prey water dependency** | **Prey group/diet** | **Adult Female weight (kg)** | **Prey size** | **In predators diet** |
| Scrub hare | *Lepus saxatilis* | Low | Other | 3 | XS | AWD CH LI |
| Sharpe's grysbok | *Raphicerus sharpei* | Low | Browser | 7.5 | S | SH |
| Slender mongoose | *Galerella sanguinea* | Low | Carnivore | 0.6 | XS | CH SH |
| Snake |  | Low | Other | 1 | XS | LE |
| Spotted hyaena | *Crocuta crocuta* | Low | Carnivore | 65 | M | LI |
| Squirrel |  | Low | Other | 0.7 | XS | LE LI SH |
| Steenbok | *Raphicerus campestris* | Low | Browser | 11 | S | AWD LE LI SH |
| Striped polecat | *Ictonyx striatus* | Low | Carnivore | 1.1 | XS | SH |
| Striped weasel | *Poecilogale albinucha* | Low | Carnivore | 0.5 | XS | LI SH |
| Tsessebe | *Damaliscus lunatus* | Moderate | Grassland grazers | 126 | L | CH |
| Vervet monkey | *Chlorocebus pygerythrus* | Moderate | Omnivorous | 4.2 | XS | LE LI SH |
| Warthog | *Phacochoerus africanus* | High | Grassland grazers | 57 | M | AWD LE LI SH |
| Waterbuck | *Kobus ellipsiprymnus* | High | Grassland grazers | 175 | L | AWD LI SH |
| White-tailed mongoose | *Ichneumia albicauda* | Low | Carnivore | 3.5 | XS | LI |
| Wildebeest | *Connochaetes taurinus* | High | Grassland grazers | 180 | L | LE LI SH |
| Zebra | *Equus quagga* | High | Grassland grazers | 302 | L | LE LI SH |

| **Table A2a. Number of samples and species in the diet of five predators in Hwange National Park, Zimbabwe.** | | | | | | |
| --- | --- | --- | --- | --- | --- | --- |
| **Predator** | **Season or Region** | | **Number of items** | **Number of scats** | | **Number of species** |
| **African wild dog** | Early Dry Wet | | 130 | 105 | | 9 |
|  | Late Dry | | 113 | 104 | | 17 |
|  | Nomadic | | 138 | 121 | | 17 |
|  | Denning | | 72 | 63 | | 8 |
|  | North East | | 100 | 89 | | 10 |
|  | North West | | 95 | 80 | | 9 |
|  | South West | | 23 | 21 | | 11 |
|  | **Total*** | | **225**  **(64 in years 2012-2015)** | **209**  **(53 in years 2012-2015)** | | **20**  **(14 in years 2012-2015)** |
| **Cheetah** | Early Dry Wet | | 14 | 15 | | 4 |
|  | Late Dry | | 11 | 11 | | 6 |
|  | **North West** | | **25** | **26** | | **7** |
| **Leopard** | Early Dry Wet | | 14 | 14 | | 9 |
|  | Late Dry | | 28 | 190 | | 10 |
|  | Nomadic | | 15 | 12 | | 8 |
|  | Breeding | | 26 | 26 | | 22 |
|  | North East | | 4 | 3 | | 4 |
|  | North West | | 39 | 35 | | 13 |
|  | South West | | 203 | 166 | | 21 |
|  | **Total** | | **246** | **204** | | **25** |
| **Lion** | Early Dry Wet | | 99 | 98 | | 26 |
|  | Late Dry | | 243 | 241 | | 30 |
|  | Nomadic | | 79 | 76 | | 20 |
|  | Breeding | | 207 | 207 | | 27 |
|  | North East | | 149 | 149 | | 23 |
|  | North West | | 140 | 138 | | 25 |
|  | South West | | 56 | 55 | | 21 |
|  | **Global*** | | **351** | **342** | | **33** |
| **Spotted hyaena** | Early Dry Wet | | 40 | 38 | | 15 |
|  | Late Dry | | 296 | 278 | | 33 |
|  | Nomadic | | 125 | 122 | | 19 |
|  | Breeding | | 86 | 81 | | 16 |
|  | North East | | 24 | 23 | | 11 |
|  | North West | | 188 | 184 | | 20 |
|  | South West | | 125 | 110 | | 27 |
|  | **Total*** | | **337** | **317** | | **33** |
| Total numbers do not necessarily match the sum of the other categories because some scat samples had missing information on location or seasonality. Numbers in red: scat samples with less than a minimum of 59 scats to describe a site species diet containing 12 or more species (Trites and Joy 2005). Analyses were not performed when there were less than 21 scats. To perform any analyses species accumulation curves needed to arrive almost to an asymptote (Appendix A, Figure A1b).  Nomadic refers to wild dogs’ nomadic season (not breeding), and breeding refers to wild dogs’ denning. Both seasons only include data on the North West and North East of Hwange National Park. | | | | | | |
| **Table A2b. Diet overlap (Pianka’s index) of African wild dogs with four predators in Hwange National Park, Zimbabwe.** | | | | | | |
|  | | **In years 2012-2015** | | | **In years 2012-2019** | |
| **Cheetah** | | **0.59** | | | **0.63** | |
| **Leopard** | | **0.61** | | | **0.63** | |
| **Lion** | | **0.74** | | | **0.71** | |
| **Spotted hyaena** | | **0.89** | | | **0.85** | |

| **Table A3a. Prey density (individuals / km^2^) in the north of Hwange National Park, Zimbabwe (2012-2015).**  Density used to calculate the diet preference of cheetahs, leopards, lions, and spotted hyaenas. | | | | | | | | | |
| --- | --- | --- | --- | --- | --- | --- | --- | --- | --- |
| **Prey species** | **North** | | | **North East** | | | **North West** | | |
|  | **Density** | **Lci** | **Uci** | **Density** | **Lci** | **Uci** | **Density** | **Lci** | **Uci** |
| Baboon  (2012-2019) | 0.51 | 0.29 | 0.89 | 0.40 | 0.18 | 0.90 | 0.61 | 0.30 | 1.23 |
| Buffalo  (2012-2017) | 3.05 | 1.82 | 5.12 | 2.09 | 1.21 | 3.62 | 4.01 | 1.97 | 8.16 |
| Duiker | 0.07 | 0.05 | 0.10 | 0.13 | 0.09 | 0.20 | 0.01 | 0.00 | 0.03 |
| Eland  (2012-2019) | 0.02 | 0.01 | 0.04 | 0.04 | 0.02 | 0.08 | 0.0003 | 0.0001 | 0.0016 |
| Elephant | 3.84 | 3.40 | 4.33 | 5.16 | 4.49 | 5.93 | 2.52 | 2.07 | 3.05 |
| Giraffe | 0.35 | 0.29 | 0.43 | 0.46 | 0.37 | 0.58 | 0.24 | 0.18 | 0.33 |
| Impala | 6.40 | 5.39 | 7.62 | 2.09 | 1.48 | 2.95 | 11.2 | 9.18 | 13.67 |
| Kudu | 1.53 | 1.28 | 1.83 | 1.42 | 1.18 | 1.70 | 1.65 | 1.29 | 2.12 |
| Reedbuck  (2012-2016) | 0.08 | 0.05 | 0.13 | 0.00 | 0.00 | 0.00 | 0.17 | 0.11 | 0.27 |
| Roan | 0.11 | 0.06 | 0.19 | 0.12 | 0.06 | 0.25 | 0.10 | 0.05 | 0.22 |
| Sable  (2012-2016) | 0.06 | 0.04 | 0.09 | 0.09 | 0.06 | 0.15 | 0.03 | 0.01 | 0.07 |
| Steenbok (2012-2019) | 0.98 | 0.86 | 1.11 | 1.87 | 1.65 | 2.12 | 0.07 | 0.05 | 0.11 |
| Warthog | 0.70 | 0.59 | 0.83 | 0.39 | 0.30 | 0.50 | 1.01 | 0.83 | 1.24 |
| Waterbuck  (2012-2017) | 0.11 | 0.08 | 0.15 | 0.08 | 0.05 | 0.13 | 0.14 | 0.09 | 0.21 |
| Wildebeest  (2012-2016) | 0.22 | 0.14 | 0.35 | 0.43 | 0.27 | 0.67 | 0.01 | 0.01 | 0.03 |
| Zebra | 1.02 | 0.85 | 1.23 | 1.31 | 1.08 | 1.60 | 0.73 | 0.52 | 1.03 |
| Lci = lower confidence interval; Uci = upper confidence interval.  All densities include both seasons. | | | | | | | | | |

| **Table A3b. Prey density (individuals / km^2^) in the north of Hwange National Park, Zimbabwe (2012-2019).**  Density used to calculate the diet preference of African wild dogs. | | | | | | | | | |
| --- | --- | --- | --- | --- | --- | --- | --- | --- | --- |
| **Prey species** | **North** | | | **North East** | | | **North West** | | |
|  | **Density** | **Lci** | **Uci** | **Density** | **Lci** | **Uci** | **Density** | **Lci** | **Uci** |
| Baboon | 0.51 | 0.29 | 0.89 | 0.40 | 0.18 | 0.90 | 0.61 | 0.30 | 1.23 |
| Duiker | 0.08 | 0.06 | 0.09 | 0.13 | 0.11 | 0.17 | 0.02 | 0.01 | 0.03 |
| Impala | 6.32 | 5.52 | 7.23 | 1.63 | 1.34 | 2.00 | 10.94 | 9.42 | 1.27 |
| Kudu | 1.12 | 0.99 | 1.27 | 1.18 | 1.04 | 1.34 | 1.07 | 0.89 | 1.28 |
| Reedbuck | 0.07 | 0.05 | 0.11 | 0.00 | 0.00 | 0.01 | 0.15 | 0.10 | 0.21 |
| Sable | 0.08 | 0.05 | 0.12 | 0.14 | 0.10 | 0.21 | 0.02 | 0.01 | 0.05 |
| Steenbok | 0.98 | 0.86 | 1.11 | 1.87 | 1.65 | 2.12 | 0.07 | 0.05 | 0.11 |
| Warthog | 0.71 | 0.62 | 0.80 | 0.38 | 0.32 | 0.46 | 1.03 | 0.89 | 1.19 |
| Waterbuck | 0.10 | 0.07 | 0.14 | 0.09 | 0.06 | 0.13 | 0.12 | 0.08 | 0.18 |
| Lci = lower confidence interval; Uci = upper confidence interval.  All densities include both seasons. | | | | | | | | | |

| **Table A4a. Average of relative abundance index (RAI) (independent records/ trap-days) of prey in Hwange National Park, Zimbabwe (2013-2015).**  RAI used to calculate the diet preference of cheetahs, leopards, lions, and spotted hyaenas. | | | | |
| --- | --- | --- | --- | --- |
| **Prey species** | **Total** | **North East**  **(Max whd)**  (2014, 2015) | **North West**  **(High whd)**  (2013, 2014) | **South West**  **(Low whd)**  (2013, 2017) |
| Aardvark | 0.35 | 0.16 | 0.56 | 0.22 |
| Baboon | 7.95 | 7.52 | 8.38 |  |
| Buffalo | 1.11 | 0.99 | 2.28 | 0.46 |
| Bushbuck | 1.36 |  | 1.36 |  |
| Bushpig | 0.09 | 0.09 |  | 0.21 |
| Duiker | 6.08 | 9.47 | 2.77 | 3.30 |
| Eland | 1.32 | 2.77 | 0.90 | 0.60 |
| Elephant | 124.28 | 166.26 | 112.17 | 90.24 |
| Gemsbok | 0.29 | 0.17 |  | 0.43 |
| Giraffe | 4.51 | 4.86 | 4.15 | 4.44 |
| Impala | 3.49 | 2.12 | 5.74 | 1.07 |
| Kudu | 2.48 | 3.21 | 2.40 | 1.01 |
| Porcupine | 4.96 | 2.09 | 8.84 | 3.07 |
| Reedbuck | 0.48 |  | 0.48 | 0.62 |
| Roan | 0.78 | 0.62 | 1.29 | 0.17 |
| Sable | 0.86 | 0.99 | 0.60 | 0.04 |
| Sharpe's grysbok | 1.11 |  | 1.11 |  |
| Steenbok | 7.27 | 10.34 | 1.57 | 8.88 |
| Tsessebe | 0.18 |  | 0.18 |  |
| Warthog | 3.01 | 1.55 | 5.03 | 4.12 |
| Waterbuck | 0.63 |  | 0.63 | 0.10 |
| Wildebeest | 1.92 | 1.92 |  | 0.34 |
| Zebra | 3.74 | 3.68 | 5.19 | 1.45 |
| RAI average of surveys performed in nine different sites shown in Figure A1.  For South West 2017 year was added to include more than only one survey in the region.  whd = waterhole density.  Blank spaces mean that there were no observations to calculate RAI. | | | | |

| **Table A4b. Average of relative abundance index (RAI) (independent records/ trap-days) of prey in Hwange National Park, Zimbabwe (2013-2019).**  RAI used to calculate the diet preference of African wild dogs. | | | | |
| --- | --- | --- | --- | --- |
| **Prey species** | **Total** | **North East**  **(Max whd)**  (2014, 2015, 2018) | **North West**  **(High whd)**  (2013, 2014, 2019) | **South West**  **(Low whd)**  (2013, 2017) |
| Baboon | 8.29 | 8.45 | 8.02 |  |
| Bat-eared fox | 0.58 | 0.76 | 0.61 | 0.19 |
| Bushbuck | 0.72 |  | 0.72 |  |
| Bushpig | 0.17 | 0.15 |  | 0.21 |
| Duiker | 5.64 | 8.40 | 2.25 | 3.30 |
| Gemsbok | 0.30 | 0.11 |  | 0.43 |
| Impala | 5.26 | 2.50 | 14.06 | 1.07 |
| Kudu | 2.50 | 2.99 | 3.17 | 1.01 |
| Porcupine | 3.67 | 1.88 | 7.27 | 3.07 |
| Reedbuck | 0.36 | 0.29 | 0.26 | 0.62 |
| Sable | 0.96 | 1.37 | 0.37 | 0.04 |
| Scrub hare | 3.28 | 1.03 | 3.87 | 6.63 |
| Steenbok | 6.90 | 9.24 | 1.81 | 8.88 |
| Warthog | 4.90 | 2.79 | 9.21 | 4.12 |
| Waterbuck | 0.53 | 0.14 | 0.81 | 0.10 |
| RAI average of surveys performed in nine different sites shown in Figure A1.  Blank spaces mean that there were no observations to calculate the relative abundance index.  whd = waterhole density. | | | | |

| **Table A5a. Seasonal diet comparisons of African wild dogs, lions and spotted hyaenas in Hwange National Park, Zimbabwe.** | | |
| --- | --- | --- |
|  | **Weather season** | **Behavioural season** |
| Season  A difference in diet of predators between seasons | pseudo-F_1_,_839_= 2.31  p = 0.79  r^2^ = 0.0027 | pseudo-F_2_,_815_= 4.95  p = 0.14  r^2^ = 0.012 |
| Predator | pseudo-F_2_,_839_= 11.76  p < 0.001*  r^2^ = 0.0027 | pseudo-F_2_,_815_= 8.83  p < 0.001*  r^2^ = 0.021 |
| Season*Predator  a difference in diet for each predator between seasons | pseudo-F_2_,_839_= 1.14  p = 0.59  r^2^ = 0.0026 | pseudo-_1_,_815_= 0.64  p = 0.16  r^2^ = 0.0017 |
| PERMANOVA results. * significant differences: p < 0.05 | | |

| **Table A5b. Regional diet differences of predators in Hwange National Park, Zimbabwe.** | | | |
| --- | --- | --- | --- |
|  | **Maximum whd (NE) vs. High whd (NW)** | **High whd (NW) vs. Low whd (SW)** | **Maximum whd (NE) vs. Low whd (SW)** |
| **African wild dog** | pseudo-F_1_,_167_= 4.14  p = 0.0039*  r^2^ = 0.024 | pseudo-F_1_,_99_= 3.59  p = 0.35  r^2^ = 0.035 | pseudo-F_1_,_108_= 2.036  p = 0.52  r^2^ = 0.018 |
|  | **All areas compared**  (as results were also non-significant when compared one area against another one) | | |
| **Leopard** | pseudo-F_2_,_201_= 3.27  p = 0.13  r^2^ = 0.031 | | |
| **Lion** | pseudo-F_2_,_339_= 2.67  p = 0.057  r^2^ = 0.016 | | |
| **Spotted hyaena** | pseudo-F_2_,_314_= 3.63  p = 0.32  r^2^ = 0.023 | | |
| PERMANOVA results.  whd= waterhole density. | | | |

| **Table A6. Differences between the diet of African wild dogs with the diet of other predators per area and per prey category in Hwange National Park, Zimbabwe.** | | | |
| --- | --- | --- | --- |
|  | **African wild dogs**  **vs.** | | |
|  | **Leopard** | **Lion** | **Spotted hyaena** |
| **Prey water dependency** | |  |  |
| Maximum whd (NE) | NA | No differences in any areas.  pseudo-F_1_,_483_= 6.91  p = 0.70  r^2^ = 0.014 | No differences in any areas.  pseudo-F_1_,_461_= 7.91  p = 0.25  r^2^ = 0.017 |
| High whd (NW) | pseudo-F_1_,_113_= 61.37  p = 0.081  r^2^ = 0.012 |  |  |
| Low whd (SW) | pseudo-F_1_,_185_= 5.27  p = 0.0069*  r^2^ = 0.028 |  |  |
| **Prey diet** | |  |  |
| Maximum whd (NE) | NA | pseudo-F_1_,_236_= 25.92  p = 0.0019*  r^2^ = 0.099 | pseudo-F_1_,_110_= 7.38  p = 0.14  r^2^ = 0.063 |
| High whd (NW) | pseudo-F_1_,_113_= 11.28  p = 0.0029*  r^2^ = 0.091 | pseudo-F_1_,_216_= 29.36  p < 0.001*  r^2^ = 0.12 | pseudo-F_1_,_262_= 20.57  p = 0.0019*  r^2^ = 0.073 |
| Low whd (SW) | pseudo-F_1_,_185_= 0.14  p = 0.65  r^2^ = 0.0026 | pseudo-F_1_,_74_= 0.069  p = 0.92  r^2^ = 0.0009 | pseudo-F_1_,_129_= 2.69  p = 0.021*  r^2^ = 0.024 |
| **Prey size** |  |  |  |
| Maximum whd (NE) | NA | pseudo-F_1_,_236_= 4.95  p = 0.042  r^2^ = 0.021 | pseudo-F_1_,_110_= 0.86  p = 0.57  r^2^ = 0.0078 |
| High whd (NW) | pseudo-F_1_,_113_= 9.98  p < 0.001*  r^2^ = 0.081 | pseudo-F_1_,_216_= 10.95  p = 0.002*  r^2^ = 0.048 | pseudo-F_1_,_262_= 8.53  p = 0.0069*  r^2^ = 0.031 |
| Low whd (SW) | pseudo-F_1_,_185_= 2.55  p = 0.071  r^2^ = 0.014 | pseudo-F_1_,_74_= 1.54  p = 0.21  r^2^ = 0.020 | pseudo-F_1_,_129_= 1.62  p = 0.20  r^2^ = 0.012 |
| whd = waterhole density.  NA = not applicable, not tested due to lack of data. | | | |

| **Table A7. Differences on prey categories in the diet of predators in Hwange National Park, Zimbabwe.** | | | |
| --- | --- | --- | --- |
|  | **Maximum whd (NE) vs. High whd (NW)** | **High whd (NW) vs. Low whd (SW)** | **Maximum whd (NE) vs. Low whd (SW)** |
| **Prey diet** | |  |  |
| African wild dog | pseudo-F_1_,_167_= 2.87  p = 0.048  r^2^ = 0.017 | pseudo-F_1_,_99_= 6.68  p = 0.52  r^2^ = 0.061 | pseudo-F_1_,_108_= 2.39  p = 0.26  r^2^ = 0.021 |
| Leopard | NA | pseudo-F_1_,_199_= 7.37  p = 0.13  r^2^ = 0.036 | NA |
| Lion | No differences in regions.  pseudo-F_2,339_= 5.52, p = 0.11, r^2^ = 0.031 | | |
| Spotted hyaena | No differences in regions.  pseudo-F_2_,_314_= 2.66, p = 0.53, r^2^ = 0.017 | | |
| **Prey size** |  |  |  |
| African wild dog | pseudo-F_1_,_167_= 4.61  p = 0.015*  r^2^ = 0.027 | pseudo-F_1_,_99_= 7.99  p = 0.29  r^2^ = 0.074 | pseudo-F_1_,_108_= 4.52  p = 0.20  r^2^ = 0.040 |
| Leopard | NA | pseudo-F_1_,_199_= 6.27  p = 0.46  r^2^ = 0.031 | NA |
| Lion | No differences in regions.  pseudo-F_2_,_339_= 2.83, p = 0.17, r^2^ = 0.016 | | |
| Spotted hyaena | No differences in regions.  pseudo-F_2_,_314_= 8.00, p = 0.37, r^2^ = 0.048 | | |
| NA = not applicable, not tested due to lack of data.  whd = waterhole density. | | | |

| **Table A8. Percentage of prey water dependency in the diet of four predators in three different regions of Hwange National Park, Zimbabwe.** | | | | | | | | | | | |
| --- | --- | --- | --- | --- | --- | --- | --- | --- | --- | --- | --- |
| **Prey water dependency** | **African wild dog** | | | **Leopard** | | **Lion** | | | **Spotted hyaena** | | |
|  | Max whd  NE | High whd NW | Low whd  SW | High whd NW | Low whd  SW | Max whd  NE | High whd NW | Low whd  SW | Max whd  NE | High whd NW | Low whd  SW |
| High | 32% | 49% | 23% | 45% | 25% | 59% | 63% | 26% | 63% | 50% | 47% |
| Moderate | 37% | 23% | 36% | 26% | 9% | 23% | 27% | 40% | 17% | 31% | 14% |
| Low | 31% | 28% | 41% | 29% | 66% | 18% | 10% | 34% | 21% | 19% | 39% |

**
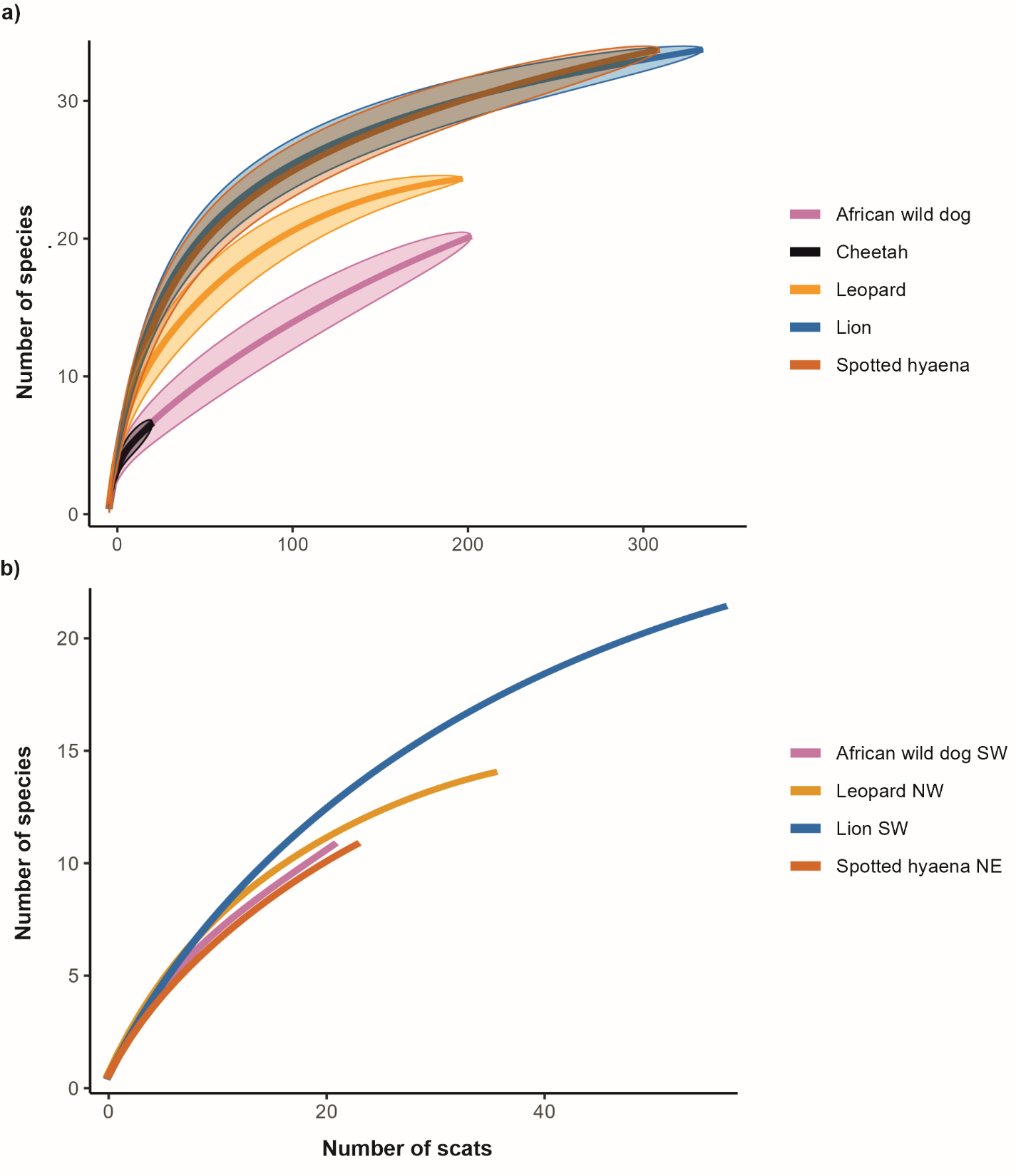
**

**Figure A1** a) Species accumulation curve of expected mean prey richness from five predator scats collected in Hwange National Park, Zimbabwe, 2012– 2019. The shadow area represents 95% confidence intervals based on 10,000 permutations. b) Species accumulation curve of expected mean prey richness from four predator scats collected in three specific areas of Hwange National Park, Zimbabwe, 2012– 2019. Areas: SW = South West; NW = North West; NE = North East.

**
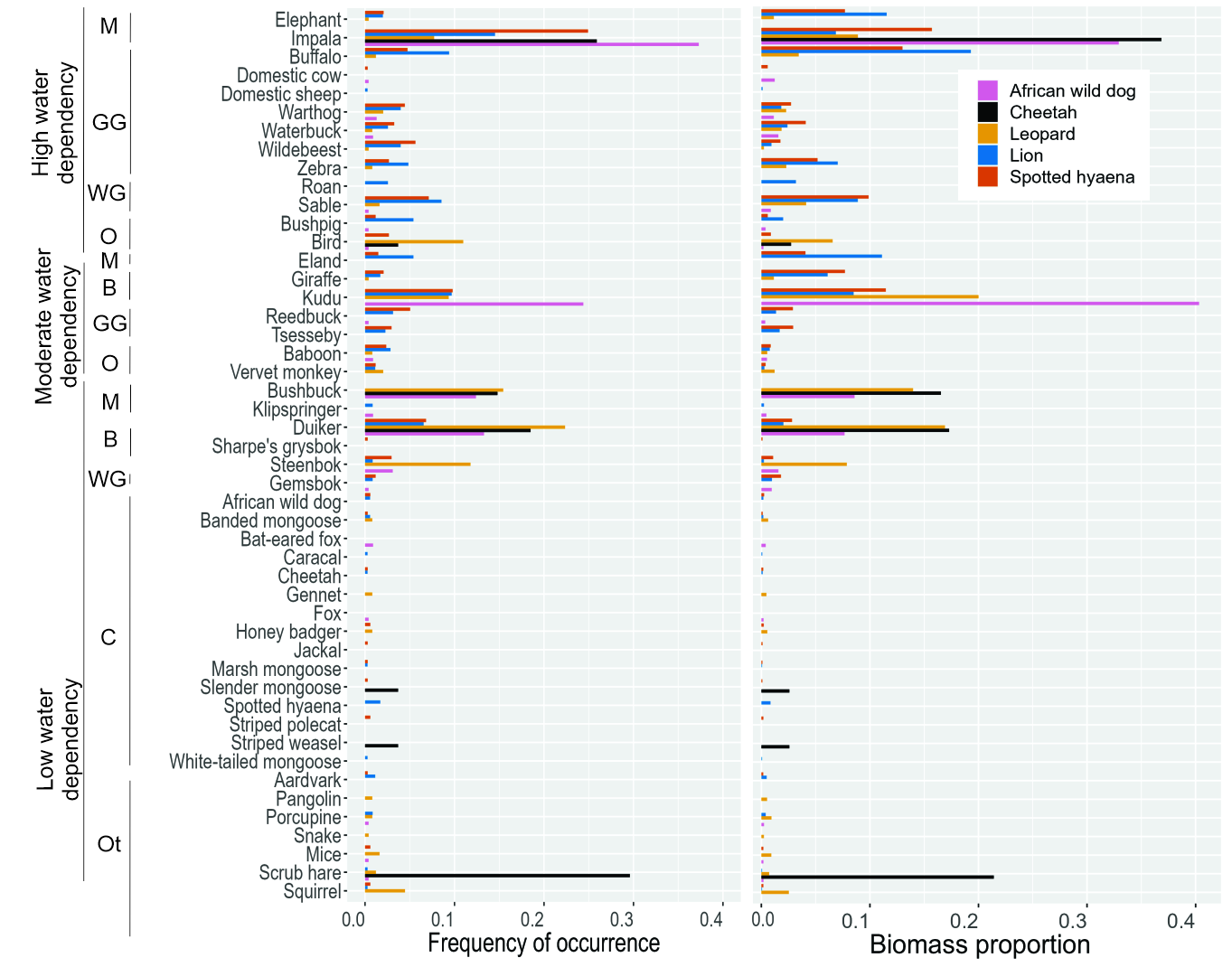
**

**Figure A2** Frequency of occurrence and biomass proportion of the diet of five predators in Hwange National Park, Zimbabwe. Prey species are ordered first by water dependency and then by prey diet: M = mixed (browser, grazer), B = browser, GG: grassland grazer, WG = woodland grazer, O = omnivorous, C = Carnivore, Ot = other.


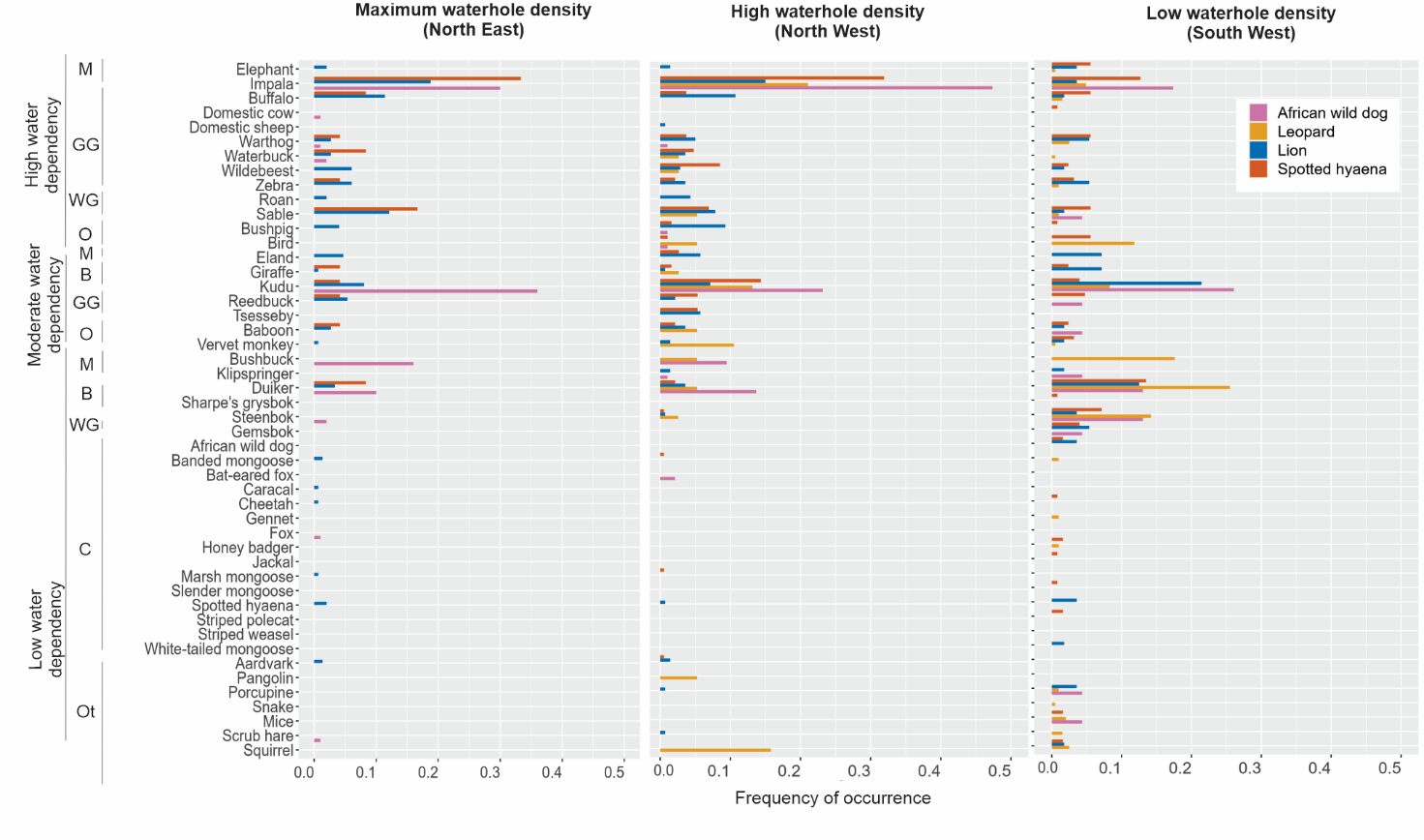


**Figure A3** Frequency of occurrence per area of the diet of five predators in three areas of Hwange National Park, Zimbabwe. Prey species are ordered first by water dependency and then by prey diet: M = mixed (browser, grazer), B = browser, GG: grassland grazer, WG = woodland grazer, O = omnivorous, C = Carnivore, Ot = other.


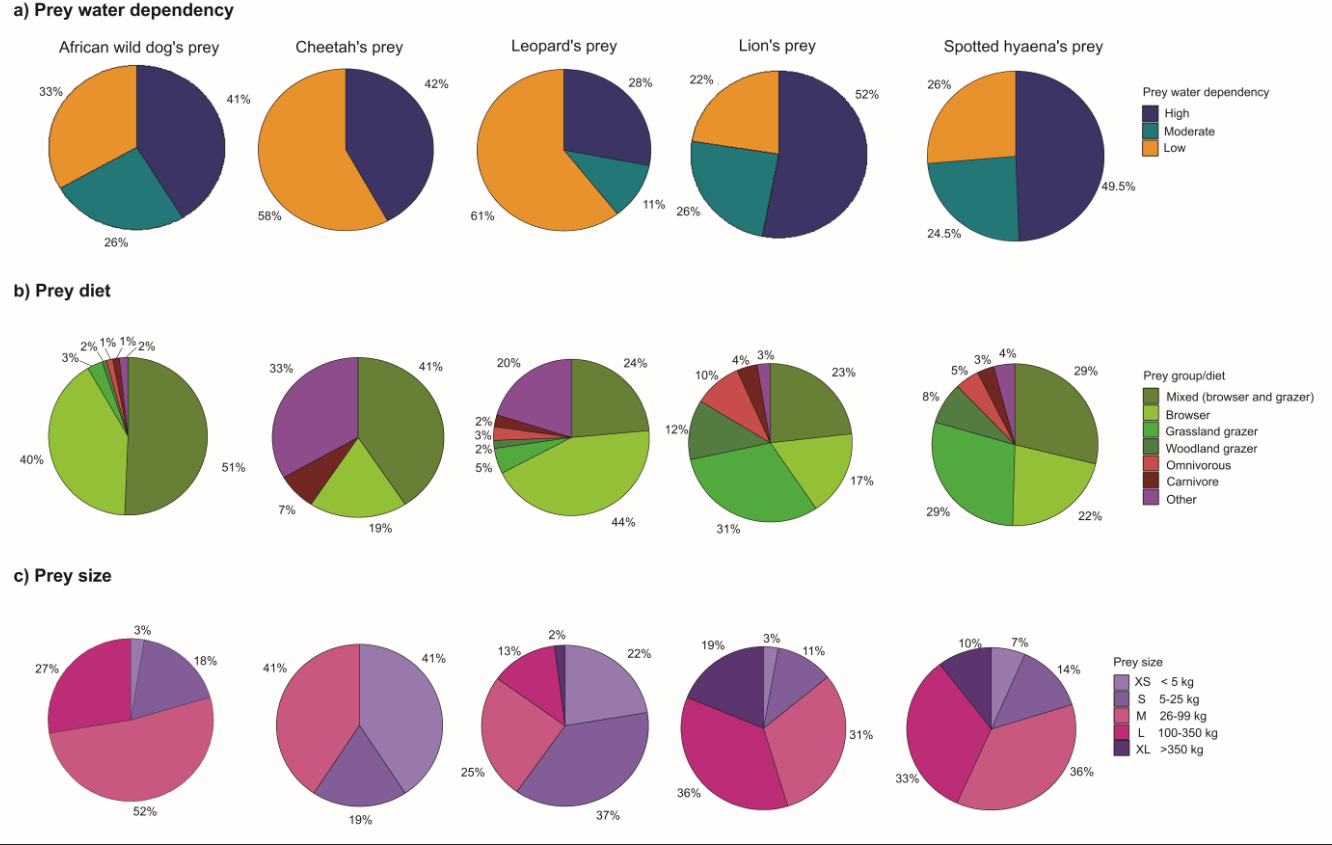


**Figure A4** Distribution of a) Prey water dependency, b) prey diet, and c) prey size of the five large predators in Hwange National Park, Zimbabwe.


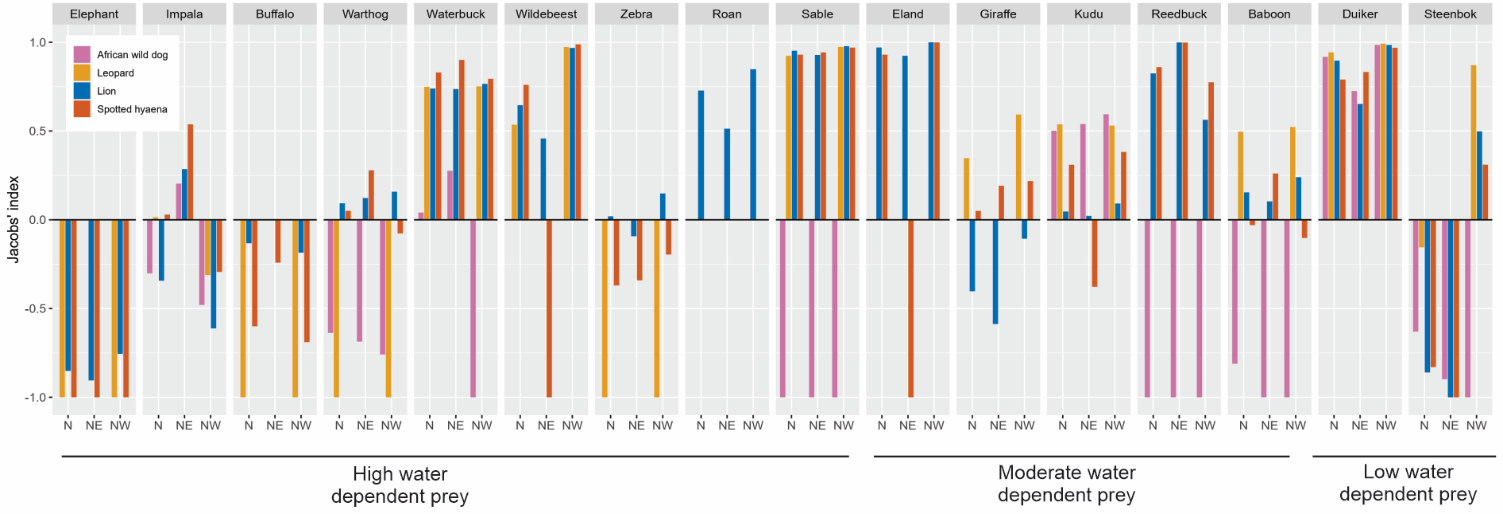


**Figure A5** Diet preference (Jacobs’ Index calculated with prey density) of five predators in Hwange National Park, Zimbabwe. N = North (only including NE and NW); NE = North East (Maximum waterhole density area); NW = North West (High waterhole density area). Cheetah not included due to little sample size.

**Supplementary references:**

Trites AW, Joy R (2005) Dietary Analysis From Fecal Samples: How Many Scats Are Enough? J Mammal 86:704–712. https://doi.org/10.1644/1545-1542(2005)086[0704:DAFFSH]2.0.CO;2
